# Visualizing Synaptic Multi-Protein Patterns of Neuronal Tissue With DNA-Assisted Single-Molecule Localization Microscopy

**DOI:** 10.3389/fnsyn.2021.671288

**Published:** 2021-06-17

**Authors:** Kaarjel K. Narayanasamy, Aleksandar Stojic, Yunqing Li, Steffen Sass, Marina R. Hesse, Nina S. Deussner-Helfmann, Marina S. Dietz, Thomas Kuner, Maja Klevanski, Mike Heilemann

**Affiliations:** ^1^Department of Functional Neuroanatomy, Institute for Anatomy and Cell Biology, Heidelberg University, Heidelberg, Germany; ^2^Institute of Physical and Theoretical Chemistry, Goethe-University Frankfurt, Frankfurt, Germany

**Keywords:** single-molecule localization microscopy, super-resolution microscopy, DNA-PAINT, neuronal synapse, multiplexing, Exchange PAINT, semi-thin brain tissue sections, tissue imaging

## Abstract

The development of super-resolution microscopy (SRM) has widened our understanding of biomolecular structure and function in biological materials. Imaging multiple targets within a single area would elucidate their spatial localization relative to the cell matrix and neighboring biomolecules, revealing multi-protein macromolecular structures and their functional co-dependencies. SRM methods are, however, limited to the number of suitable fluorophores that can be imaged during a single acquisition as well as the loss of antigens during antibody washing and restaining for organic dye multiplexing. We report the visualization of multiple protein targets within the pre- and postsynapse in 350–400 nm thick neuronal tissue sections using DNA-assisted single-molecule localization microscopy (SMLM). In a single labeling step, antibodies conjugated with short DNA oligonucleotides visualized multiple targets by sequential exchange of fluorophore-labeled complementary oligonucleotides present in the imaging buffer. This approach avoids potential effects on structural integrity when using multiple rounds of immunolabeling and eliminates chromatic aberration, because all targets are imaged using a single excitation laser wavelength. This method proved robust for multi-target imaging in semi-thin tissue sections with a lateral resolution better than 25 nm, paving the way toward structural cell biology with single-molecule SRM.

## Introduction

Super-resolution microscopy (SRM) has revolutionized our understanding of cell biology. Single-molecule localization microscopy (SMLM) is one branch of SRM, which employs photoswitchable or transiently binding fluorophore labels and has demonstrated a near-molecular spatial resolution (Sauer and Heilemann, 2017) allowing molecular quantification (Dietz and Heilemann, 2019). A further exciting development was the integration of short DNA oligonucleotides into the concept of SMLM, as realized in DNA point accumulation in nanoscale topography (DNA-PAINT) (Jungmann et al., 2010). The short oligonucleotides act as transiently hybridizing pairs, with one coupled to a target protein (the “docking strand”, attached to e.g., an antibody) and a second carrying a fluorophore (the “imager strand”) suspended in the imaging buffer. The transient hybridization of both oligonucleotides generates a temporally short and spatially localized signal, which at low concentration of imager strands is recorded as a single-molecule emission event. A particular strength of DNA-PAINT is that multi-color imaging is not limited by the number of fluorophores that can be separated by their emission spectra, but instead the “color” is encoded into the DNA sequence of the pair of docking and imager strand utilized in consecutive imaging rounds. Implementing an experimental protocol that exchanges imager strands in the buffer solution allows for imaging of more targets than if discrimination occurs on the basis of emission spectra, a method termed Exchange PAINT (Jungmann et al., 2014). Multiplexing and the excellent spatial resolution achieved with DNA-PAINT is now beginning to evolve as a tool in cell biology (Harwardt et al., 2020; Schröder et al., 2020; Strauss and Jungmann, 2020).

The next important step in the application of SRM to cell biology is to visualize the nano-architecture of proteins in the functional context, which demands for super-resolution imaging in tissue and multiplexed imaging of many proteins in the same sample. SMLM imaging of 15 protein targets in cells and tissue was recently achieved using multiple rounds of antibody labeling and fluorophore staining (Klevanski et al., 2020). Here, we demonstrate the integration of DNA-PAINT for super-resolution imaging of structurally preserved neuronal brain tissue from rats, and we achieve a lateral spatial resolution of better than 25 nm. We demonstrate multiplexed imaging of four targets using only one excitation laser light source and the same fluorophore for all targets. This advantage further demonstrates the robustness of Exchange PAINT as multiple structures can be aligned without the need for chromatic correction. In addition, a single antibody labeling step minimizes sample damage that might occur with many repeated immunostainings. Furthermore, we integrate recent developments in DNA-PAINT labels that allow for faster imaging (Strauss and Jungmann, 2020). In short, we established an experimental pipeline for robust and fast super-resolution imaging of proteins in structurally preserved tissue that achieves near-molecular spatial resolution and enables the ultrastructural investigation of protein assemblies in their native environment.

## Results

**FIGURE 1:**
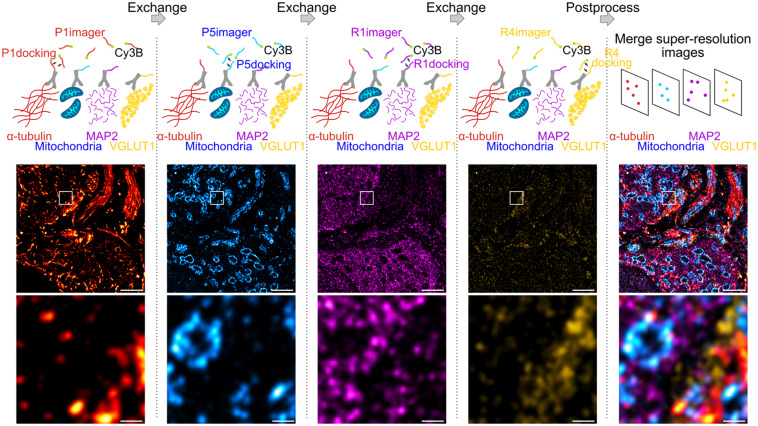
Exchange DNA-PAINT of four targets imaged sequentially. Four protein targets in tissue were labeled with primary antibodies and their corresponding secondary antibody-docking strand conjugate (P1, P5, R1, or R4). The Cy3B labeled imager strands were imaged sequentially by strand type with wash steps between each imaging round. All SMLM rendered images depicting each target were merged to obtain a multi-protein super-resolved image. Scale bar 1 μm **(top)** and 0.1 μm **(bottom)**.

We employed Exchange PAINT (Jungmann et al., 2014) for super-resolution imaging of multiple protein targets in neuronal tissue. Using this technique, four proteins were immunolabeled simultaneously, thereby maintaining low sample preparation time while obtaining an information-rich dataset. In a first experiment, α-tubulin, mitochondria (TOM20), microtubule-associated protein 2 (MAP2), and vesicular glutamate transporter (VGLUT1) were labeled with primary antibodies (Ab) and secondary Ab conjugated to DNA docking strands (P1, P5, R1, or R4; see section “Materials and Methods”) (Figure 1). Docking and imager strand sequences and modifications are reported in Table 1 and Table 2, respectively.

**TABLE 1:** Sequences of docking strands.

Name	Sequence	Modification
P1 docking strand	TTATACATCTA	5′ – Thiol
P5 docking strand	TTTCAATGTAT	5′ – Thiol
R1 docking strand	TCCTCCTCCTCCTCCTCCT	5′ – Azide
R4 docking strand	ACACACACACACACACACA	5′ – Azide

**TABLE 2:** Sequences of imager strands.

Name	Sequence	Modification
P1 imager strand	TAGATGTAT	3′ – Cy3B
P5 imager strand	CATACATTGA	3′ – Cy3B
R1 imager strand	AGGAGGA	3′ – Cy3B
R4 imager strand	TGTGTGT	3′ – Cy3B

The protocol for sequential DNA-PAINT imaging started by adding P1 imager strands into the buffer and imaging α-tubulin in the first round, followed by washing away the strands and replacing them with P5 imager strands for mitochondrial imaging. This set of steps was repeated with R1 and R4 strands until all labeled proteins were imaged within the same region of interest (ROI). Each set of frames was rendered individually and merged together using fiducial markers to obtain an overlay of four protein targets organized within tissue (see section “Materials and Methods”).

**FIGURE 2:**
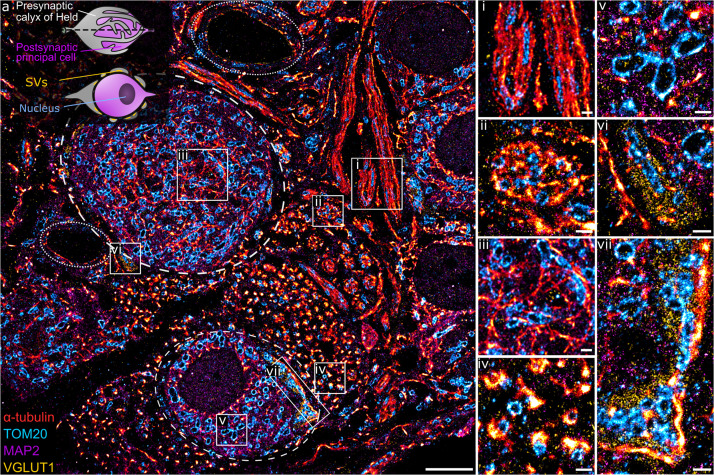
**(a)** A four-target overlay DNA-PAINT image of MNTB tissue with two calyx synapses and corresponding postsynaptic principal cell (stippled lines), capillaries (dotted lines), and a graphical representation of the calyx of Held (inset). **(i–vii)** Magnification of regions within the primary image **(a)** showing different protein morphologies and organization of tubulin, mitochondria, MAP2, and VGLUT1 within the MNTB. Scale bar 5 μm **(a)** and 0.5 μm **(i–vii)**.

This method was implemented to study the structure and organization of proteins in semi-thin neuronal tissue sections, specifically within the medial nucleus of the trapezoid body (MNTB) region, which contains the calyx of Held (Figure 2a, inset), a giant presynaptic terminal (gray) partially enveloping the postsynaptic principal cell (purple) with finger-like protrusions. Each calyx contains hundreds of active zones (AZs) for glutamatergic synaptic transmission (Sätzler et al., 2002; Dondzillo et al., 2010). A transverse section of the calyx of Held reveals the soma of the principal cell and presynaptic endings distributed around the edges, exposing the AZs of the synaptic contact. α-tubulin, mitochondria, MAP2, and VGLUT1 were stained with the Ab-DNA conjugate and imaged with Exchange PAINT (Figure 2a). The image shows several principal cells enveloped by the presynaptic calyx of Held, two of them fully visible within the tissue matrix (stippled lines), with one sectioned across the nucleus, as well as axons and capillaries (dotted line). MAP2 is commonly used as a neuronal marker as it selectively labels neuronal cells, specifically the cytoplasm of the soma and dendrites (Sarnat, 2013). VGLUT1 is a marker for synaptic vesicles (SVs), which are concentrated in the presynaptic terminal of the calyx. Regions with interesting morphological and organizational protein distribution are magnified in Figures 2i–iv, representing the co-organization between tubulin (red) and mitochondria (cyan) within morphologically distinct structures. Figures 2i,ii represent the transverse- and cross-sections of axons, respectively, which show the parallel organization of tubulin filaments along the length of the axon or the circular arrangement of tubulin within an axon bundle. Mitochondria within the axons are thin, elongated structures sandwiched between tubulin filaments and are distributed randomly along and across the axon bundle. The protein organization seen here is in line with the fact that tubulin filaments (microtubules) play a role in mitochondrial transport along axons to the presynaptic terminals where they are needed to maintain continuous synaptic transmission (Verstreken et al., 2005; Zorgniotti et al., 2021).

Apart from axons, tubulin and mitochondria are also co-organized in other parts of the neural network. Figure 2iii shows the organization between tubulin and mitochondria within the soma of the principal cell. Here, tubulin filaments appear as short, thin fibrils without a distinct organizational pattern. Similarly, mitochondria show random arrangement within the soma. MAP2 clearly labels the soma of principal cells with larger and oval shaped mitochondria embedded within the matrix (Figure 2v). Another morphologically distinct structure of tubulin is observed next to the smaller calyx synapse. Here, tubulin forms dense, small bundles and each bundle is organized tightly with 1–2 mitochondria (Figure 2iv).

Figures 2vi,vii show presynaptic compartments of the calyces containing SV clusters (yellow) next to the principal cell. A feature of interest is the proximity of SVs to tubulin, which can be found as punctate structures embedded in the synaptic site (Figure 2vi) or bordering the outer edge of the SV cluster (Figure 2vii). The close proximity of tubulin and SVs has been documented before (Piriya Ananda Babu et al., 2020) and function in the transport and regulation of SV precursors to the presynaptic terminal. Furthermore, mitochondria localized in between SVs in the presynapse are morphologically more compact and dense compared to those in the principal cell.

**FIGURE 3:**
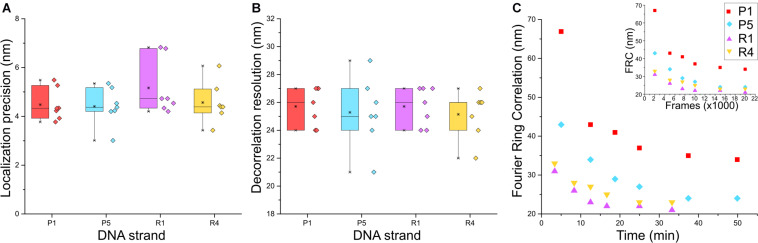
Comparison between P1, P5, R1, and R4 DNA-PAINT strands for **(A)** localization precision by nearest neighbor analysis (Endesfelder et al., 2014) and **(B)** rendered image resolution by decorrelation analysis; n = 7. **(C)** Fourier Ring Correlation (FRC) resolution trend of the four strands over image acquisition time and FRC over number of frames (inset); *n* = 1.

Next, we characterized the image quality using experimental parameters used for SMLM data (Sauer and Heilemann, 2017). We determined the localization precision and the spatial resolution achieved with the different imager strands used in the Exchange PAINT experiment, i.e., P1, P5, R1, and R4. The P1 and P5 strands were among the first DNA sequences used in DNA-PAINT and hybridized into a duplex of nine nucleotide base pairs (Schnitzbauer et al., 2017). The R1 and R4 docking strands contained repeated and concatenated sequences that allowed the hybridization of multiple imager strands onto one docking strand increasing the frequency of events (Strauss and Jungmann, 2020). The localization precision of events was calculated from the nearest neighbor value (Endesfelder et al., 2014; Figure 3A) and the lowest localization precision value obtained was 3 nm with P5 strands. Median values recorded for all four strands were below 5 nm. The spatial resolution obtained for the four imager strands was determined by a decorrelation analysis (Descloux et al., 2019) which reported median values around 25 nm, and the highest resolution achieved was 21 nm for the P5 strand (Figure 3B).

Although there was no apparent difference in the localization precision and resolution between the P strands and R strands, a marked advantage of the R strands was the shorter acquisition time required during imaging and increased frequency of binding between imager and docking strands, which was reported to reduce the imaging time (Strauss and Jungmann, 2020). We sought to quantify this using Fourier Ring Correlation (FRC) analysis (Nieuwenhuizen et al., 2013) by calculating the resolution of images formed over time. Each super-resolved image was reconstructed from 20,000 frames with an integration time of 150 ms (P1 and P5) or 100 ms (R1 and R4), respectively. Figure 3C shows that the FRC curve plateaued before imaging time was complete, therefore all images were able to achieve maximum resolution at 20 000 frames. Saturation of resolution was calculated at 95% of the lowest resolution value achieved for each image. Indeed, both R strands were able to achieve maximum resolution faster than P strands, with R1 and R4 at 17 and 20 min, and P1 and P5 at 37 and 34 min, respectively. The reduction in imaging time by 15–20 min, and comparable localization precision and resolution make the R strands suitable for faster Exchange PAINT imaging of multiple targets.

**FIGURE 4:**
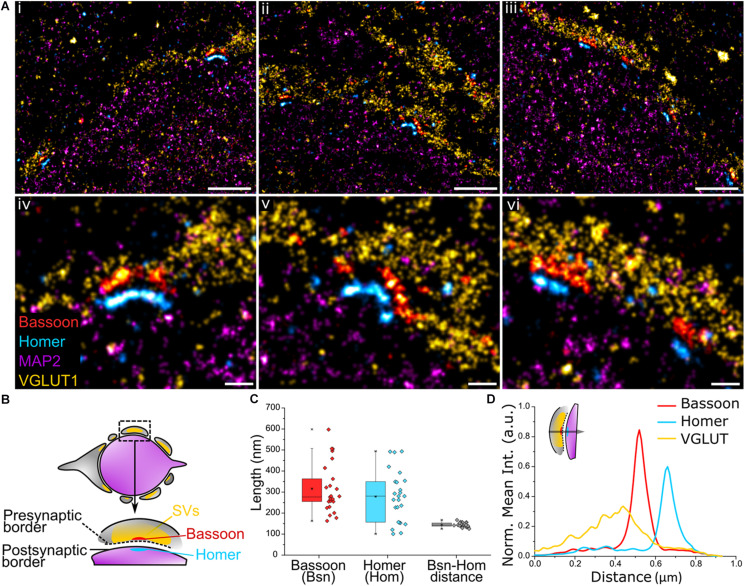
**(A)** Four-target images of **(i–iii)** the organization of multiple Bassoon and Homer structures sandwiched between VGLUT1 (SV) and MAP2 (microtubules) along the presynaptic border of the calyx of Held and the postsynaptic border of the principal cell. **(iv–vi)** Magnification of the AZ-PSD interface with aligned Bassoon and Homer structures showing linear or curved morphologies. **(B)** Graphical representation of a trans-section of a calyx of Held principal cell (purple) surrounded by the presynaptic cell (gray) and the organization of Bassoon, Homer, and SVs. **(C)** Quantification of the length of Bassoon- or Homer-positive areas, and the distance between Bassoon and Homer; *n* = 25. **(D)** Line profile of 2D spatial organization of protein density based on fluorescence intensity from VGLUT1 to Bassoon to Homer; *n* = 16. Scale bar 1 μm **(i–iii)** and 0.2 μm **(iv–vi)**.

We next sought to apply Exchange PAINT to visualize a key component of the synaptic architecture – the AZ. Here, synaptic scaffold proteins Bassoon and Homer that delineate the active zone and postsynaptic density (PSD) were imaged in MNTB tissue to observe their distribution. The presynaptic region was identified using the SV marker VGLUT1 and the postsynaptic area using the neuronal marker MAP2. Multiple Bassoon (AZ) and Homer (PSD) structures represent synaptic contacts formed by the calyx and principal cell (Figure 4Ai–iii). Bassoon is located on the inner presynaptic border, defined here by the inner edge of the VGLUT1 band, and Homer is juxtaposed against Bassoon and found on the edge of the MAP2 signal (Figures 4Aiv–vi,B). Magnified images of Bassoon and Homer show highly resolved edges and a defined space in between, partially reflecting the presence of the synaptic cleft, as well as curved (Figures 4Aiv,v) or linear morphologies (Figure 4Avi) of the AZ and PSD.

The profile views of Bassoon and Homer were measured lengthwise (Figure 4C) and had a comparable median length of 277 and 281 nm, respectively (Bassoon mean = 316 nm, SD = 117; Homer mean = 278 nm, SD = 120). Of considerable interest in studies of synaptic specializations is the distance between Bassoon and Homer. These scaffold proteins are located below their respective synaptic plasma membranes, therefore, unlike the synaptic cleft which has a distance of only 28 ± 9 nm in the calyx (Sätzler et al., 2002), are spaced far enough apart to be easily resolved using super-resolution light microscopy. The Bassoon-Homer distance was found to fall into a narrow distribution range with median 143 nm (mean = 144 nm, SD = 10 nm; Figure 4C). This distance is reflected in the intensity line profiles of Bassoon and Homer (Figure 4D). This value is in good agreement with previous SMLM studies reporting values of ∼150 ± 20 nm in brain tissue (Dani et al., 2010) and ∼165 ± 9 nm in neuronal cultures (Glebov et al., 2016).

The relative localization of VGLUT1 and Homer to Bassoon was determined by measuring the fluorescence intensity profile of the corresponding proteins within the AZ from the presynaptic terminal toward the principal cell (Figure 4D and inset). The 2D line profile shows defined Bassoon and Homer peaks with respective widths of 82 nm and 85 nm at FWHM (Gaussian fitting). SVs are found to be anchored at higher density closer to Bassoon. SVs function in the release of neurotransmitters at the presynaptic AZ, hence are present in high density on the presynaptic membrane. While the exact function of Bassoon is still unknown, it was shown to play a role in short-term SV replenishment during neurotransmission (Dani et al., 2010; Hallermann et al., 2010; Parthier et al., 2018) and SV tethering to the AZ (Mukherjee et al., 2010), thus accounting for the colocalization of SVs and Bassoon.

## Discussion

Studies in structural biology require imaging in greater spatial resolution and to observe proteins in their native environment. One of the challenges in imaging neuronal structures is studying the precise organization of proteins within a dense spatial matrix as well as their relative localization to other neuronal proteins. To this end, SRM has been used as a tool due to its ability to resolve structures in the nanoscale and image multiple targets to obtain an overview of protein arrangement within neurons (Colnaghi et al., 2019; Kubo et al., 2019), and has shed light on disease pathologies within dense structures (Shahmoradian et al., 2019).

Single-molecule localization microscopy methods such as STORM and Bayesian blinking and bleaching (3B) have been used to study the organization of proteins in the AZ (Dani et al., 2010; Glebov et al., 2016). However, the number of spectrally distinct fluorophores that can be used for photoswitching and for which chromatic aberration can be corrected are limited, which prevents the imaging of more than three structures at a time. To overcome this, super-resolution imaging with dSTORM (Heilemann et al., 2008) was accomplished by sequential staining realized via bleaching, elution, and restaining using antibodies or other labels against 16 protein targets to obtain an overview of protein distribution within the calyx of Held (Klevanski et al., 2020). The signal density of a target protein can be enhanced by implementing multiple rounds of labeling and imaging (Venkataramani et al., 2018). An alternative solution to visualize protein targets in SRM is the integration of DNA-based protein labels (e.g., antibodies), such as in DNA-PAINT (Schnitzbauer et al., 2017), in which the specificity of a target is encoded in the DNA sequence attached to the protein label and probed by a sequence-complementary and fluorophore-labeled DNA oligonucleotide contained in the imaging buffer. This concept has the additional advantage of providing a nearly constant signal over time and being less prone to photobleaching, which has also been adapted to other super-resolution imaging techniques (Spahn et al., 2019a,b; Glogger et al., 2020).

DNA-PAINT can be extended to image multi-protein targets without requiring specialized optics in a concept termed Exchange PAINT (Jungmann et al., 2014). This method has previously been used to study multiple targets within primary neuronal cultures (Wang et al., 2017; Guo et al., 2019). However, to our knowledge DNA-PAINT has so far not been employed to study synaptic organization in neuronal tissue. Here, we have demonstrated the robustness of the Exchange PAINT method to image protein organization within the calyx of Held and principal cell in semi-thin MNTB tissue in super resolution. This method allows the imaging of multiple targets within a dense structure and is not limited by fluorophore type. Instead, the use of a single fluorophore type prevents chromatic aberration which allows the study of spatial arrangement of structures with better accuracy. Furthermore, Exchange PAINT does not require the use of harsh and time-consuming elution steps or bleaching methods. The single antibody labeling step for multiple target proteins reduces sample preparation time and is only limited by the availability of secondary antibody species. Further increasing the number of protein targets for multiplexing is also possible by using DNA docking strands directly conjugated to primary antibodies, extending the versatility of this method. In addition, we employed an imaging buffer with increased salinity that we reasoned stabilizes DNA duplex formation, which is in line with previous reports (Schueder et al., 2019). Using this buffer, we detected a higher number of binding events over time with the same imager strand concentration, which reduces acquisition time and maintains low background signal. At the same time, we verified that this imaging buffer does not alter the structural integrity of the tissue sample at the level of spatial resolution we attain with the imaging method. Furthermore, the use of R strands speeds up image acquisition and offers exemplary image resolution and localization precision. Indeed, the resolution achieved here surpasses that achieved in similar tissue sections with dSTORM imaging by ∼5 nm (Klevanski et al., 2020). Using Exchange PAINT, multiple dense nanostructures of the pre- and post-synapse can be super-resolved to study their nanoscale spatial patterns within structurally preserved tissue sections. A possible extension would be to incorporate quantitative DNA-PAINT into this workflow, which was recently used to determine the copy numbers of AMPA receptors (Böger et al., 2019).

In conclusion, the method presented here for multi-target imaging using Exchange PAINT in tissue represents an important step forward in studying the protein organization of synapses at the nanoscale. While studying synaptic organization in cultured cells using DNA-PAINT has been reported, it does not necessarily exemplify their native organization in tissue. Therefore, this workflow represents a means to advance the field of synaptic biology by studying structurally relevant neuronal organization *in situ* with near-molecular spatial resolution using optical SRM.

## Materials and Methods

### Medial Nucleus of the Trapezoid Body Tissue Preparation

All experiments that involved the use of animals were performed in compliance with the relevant laws and institutional guidelines of Baden–Württemberg, Germany (protocol G-75/15). Animals were kept under environmentally controlled conditions in the absence of pathogens and *ad libitum* access to food and water. Preparation of brain sections containing the MNTB for Exchange PAINT was performed according to an established protocol (Klevanski et al., 2020) with slight modifications. Briefly, Sprague-Dawley rats (Charles River) at postnatal day 13 were anaesthetized and perfused transcardially with PBS followed by 4% PFA (Sigma). Brains were dissected and further fixed in 4% PFA overnight at 4°C. On the following day 200 μm thick vibratome (SLICER HR2, Sigmann-Elektronik) sections of the brainstem (containing MNTB) were prepared. MNTB were excised and infiltrated in 2.1 M sucrose (Sigma) in 0.1 M cacodylate buffer overnight at 4°C. Tissue was mounted on a holder, plunge-frozen in liquid nitrogen in 2.1 M sucrose and semi-thin sections (350 nm) were cut using the cryo-ultramicrotome (UC6, Leica). Sections were picked up with a custom made metal loop in a droplet of 1% methylcellulose and 1.15 M sucrose and transferred to 35 mm glass bottom dishes (MatTek) pre-coated with 30 μg/ml of fibronectin from human plasma (Sigma) and TetraSpeck fluorescent beads (1:500, Invitrogen). Dishes containing sections were stored at 4°C prior to their use.

### Antibody-DNA Conjugation

Secondary antibodies of donkey anti-chicken (703-005-155), donkey anti-goat (705-005-147), donkey anti-mouse (715-005-151), and donkey anti-rabbit (711-005-152) were purchased from Jackson Immunoresearch. DNA strands were purchased from Metabion with a thiol modification on the 5′ end for each docking strand and a Cy3B dye on the 3′ end for the imager strands.

The secondary antibody to DNA docking strand conjugation was prepared using a maleimide linker as previously reported in detail (1). The thiolated DNA strands were reduced using 250 mM DTT (A39255, Thermo). The reduced DNA was purified using a Nap-5 column (17085301, GE Healthcare) to remove DTT and concentrated with a 3 kDa Amicon spin column (UFC500396, Merck Milipore).

Antibodies (>1.5 mg/mL) were reacted with the maleimide-PEG2-succinimidyl ester crosslinker in a 1:10 molar ratio and purified with 7K cutoff Zeba desalting spin columns (89882, Thermo Fisher Scientific) and concentrated to > 1.5 mg/mL. The DNA and antibody solutions were cross-reacted at a 10:1 molar ratio overnight and excess DNA was filtered through a 100 kDa Amicon spin column (UFC510096, Merck Milipore). The antibody-DNA solution was stored at 4°C.

### Immunolabeling

Tissue samples were labeled with antibodies against α-tubulin-mouse (T6199, Sigma), TOM20-rabbit (sc-11415, Santa Cruz), MAP2-chicken (188006, SySy), VGLUT1-goat (135307, SySy), Homer1/2/3-rabbit (160103, SySy), and Bassoon-mouse (SAP7F407, Enzo Life Sciences). Tissue samples in dishes were washed with PBS three times for 10 min each to remove the sucrose-methylcellulose layer and blocked with 5% fetal calf serum (FCS) for 30 min. The primary antibodies were diluted in 0.5% FCS and applied to the tissue section for 1 h at room temperature (rt) and washed off three times with PBS. The conjugated secondary antibody-DNA docking strand in 0.5% FCS was applied onto tissue for 1 h at rt and washed 3 times with PBS. The tissue was then stained with Alexa Fluor 488-conjugated WGA (WGA-A488) (W11261, Thermo Fisher Scientific) in PBS for 10 min and washed off three times with PBS.

### Image Acquisition

Single-molecule localization microscopy and widefield microscopy were performed on a modified Olympus IX81 inverted microscope setup with an Olympus 150x TIRF oil immersion objective (UIS2, 1.49NA) and the samples were illuminated in TIRF mode during acquisition. For imaging Cy3B DNA imager strands, a 561 nm laser line (Coherent Sapphire LP) was focused onto the sample at a density of 0.88 kW/cm^2^ through a 4L TIRF filter (TRF89902-EM, Chroma) and ET605/70 M nm bandpass filter (Chroma) and signals were detected with an Andor iXon EM+ DU-897 EMCCD camera (Oxford Instruments). WGA-A488 widefield images were obtained using a 491 nm laser line (Olympus Digital Laser System). SMLM frames were acquired using the multi-dimensional acquisition (MDA) mode in Micro-Manager 2.0 (Edelstein et al., 2014).

### Imaging Conditions

DNA-PAINT imaging was performed in 5× Buffer C (2.5 M NaCl; S7653, Sigma in 5x PBS; 14200-059, Gibco) supplemented with 1 mM ethylenediaminetetraacetic acid (EDTA; E6758, Sigma), 2.5 mM 3,4-dihydroxybenzoic acid (PCA; 03930590, Sigma), 10 nM protocatechuate 3,4-dioxygenase pseudomonas (PCD; P8279, Sigma), and 1 mM ( ± )-6-hydroxy-2,5,7,8-tetra-methylchromane-2-carboxylic acid (Trolox; 238813-5G, Sigma). P strands (P1 and P5) were imaged at an imager strand concentration of 0.5 nM and acquisition rate of 150 ms, and R strands (R1 and R4) at a concentration of 50 pM and acquisition rate of 100 ms. All images were acquired with 50 EM gain, for 10,000 to 20,000 frames. Exchange PAINT was performed manually by adding the imaging buffer to the sample chamber and acquiring camera images. The buffer was then removed and the sample washed five times with 1× PBS to remove all imager strands. The subsequent imaging buffer containing another imager strand was then added and the procedure repeated until all targets were imaged.

### Image Processing

Frames containing single molecule events were processed and rendered using Picasso software (Schnitzbauer et al., 2017). Events in each frame were localized by fitting using the Maximum Likelihood Estimation for Integrated Gaussian parameters (Smith et al., 2010). The localized events were then filtered by their width and height of the Point Spread Function (sx. sy). The resulting localizations were drift corrected using redundant cross-correlation (RCC), rendered using the “One Pixel Blur” function and further processed using the “linked localizations” function to merge localizations that appeared in multiple consecutive frames. Images were merged in Fiji (Schindelin et al., 2012) using the “merge channels” tool and aligned by linear transformation using 0.1 μm Tetraspeck fiducial markers (2155302, Invitrogen) as registration reference. The individual channels were assigned pseudocolours. The localization precision was determined via a nearest neighbor analysis (NeNA) (Endesfelder et al., 2014) embedded into the Picasso software. The lateral spatial resolution was calculated for rendered SMLM images using an ImageJ plugin for decorrelation analysis (Descloux et al., 2019).

### Image Analysis

The length of Bassoon and Homer were measured in ImageJ by creating a binary mask of the rendered image with the preset “moments” threshold. A line was drawn along the long axis of the AZ and PSD structure, respectively, and the length was measured. The distance between Bassoon and Homer was measured by drawing a line perpendicular to both structures and adjusting the spline fit to incorporate the linear length of the structures. The fluorescence intensity for each structure was plotted and fitted with a Gaussian function. The distance was calculated from the distance between the peak intensities of the two structures. Similarly, the line profile of Bassoon, Homer, and VGLUT1 was obtained by measuring their fluorescence intensity using the line tool with spline fit perpendicular to the structures. Fluorescence intensity against distance was averaged for all ROIs with Bassoon peak intensity as the reference point.

Fourier Ring Correlation analysis (Nieuwenhuizen et al., 2013) was performed by saving filtered and drift-corrected DNA-PAINT localizations from Picasso and opening the localizations in ThunderSTORM (Ovesný et al., 2014). Localizations were filtered according to frame length from 0 to 20,000 and each frame length was filtered into blocks of 100. Rendered images were saved and FRC values were calculated using the BIOP.FRC plugin in ImageJ with the Fixed 1/7 criteria.

## Data Availability Statement

The raw data supporting the conclusions of this article will be made available by the authors, without undue reservation.

## Ethics Statement

The animal study was reviewed and approved by the Regierungspräsidium Karlsruhe.

## Author Contributions

MH conceptualized the study. KN, MH, MK, and TK conceived the experiments. MH, KN, SS, and MK done the optical instrument set up. KN, AS, YL, MD, ND-H, MK, and MRH performed the experiments. KN, ND-H, MD, MH, and MK performed the data analysis. All authors contributed to manuscript revision, read, and approved the final submitted version.

## Conflict of Interest

The authors declare that the research was conducted in the absence of any commercial or financial relationships that could be construed as a potential conflict of interest.

## References

[B1] Böger C. Hafner A. S. Schlichthärle T. Strauss M. T. Malkusch S. Endesfelder U. (2019). Super-Resolution imaging and estimation of protein copy numbers at single synapses with DNA-point accumulation for imaging in nanoscale topography. *Neurophotonics* 6:035008. 10.1117/1.NPh.6.3.035008 PMC6795074 31637284

[B2] Colnaghi L. Russo L. Natale C. Restelli E. Cagnotto A. Salmona M. (2019). Super resolution microscopy of SUMO proteins in neurons. *Front. Cell. Neurosci.* 13:486. 10.3389/fncel.2019.00486 31749687 PMC6844275

[B3] Dani A. Huang B. Bergan J. Dulac C. Zhuang X. (2010). Superresolution imaging of chemical synapses in the brain. *Neuron* 68 843–856. 10.1016/j.neuron.2010.11.021 21144999 PMC3057101

[B4] Descloux A. Grußmayer K. S. Radenovic A. (2019). Parameter-free image resolution estimation based on decorrelation analysis. *Nat. Methods* 16 918–924. 10.1038/s41592-019-0515-7 31451766

[B5] Dietz M. S. Heilemann M. (2019). Optical super-resolution microscopy unravels the molecular composition of functional protein complexes. *Nanoscale* 11 17981–17991. 10.1039/c9nr06364a 31573593

[B6] Dondzillo A. Sätzler K. Horstmann H. Altrock W. D. Gundelfinger E. D. Kuner T. (2010). Targeted three-dimensional immunohistochemistry reveals localization of presynaptic proteins bassoon and piccolo in the rat calyx of held before and after the onset of hearing. *J. Comp. Neurol.* 518 1008–1029. 10.1002/cne.22260 20127803

[B7] Edelstein A. D. Tsuchida M. A. Amodaj N. Pinkard H. Vale R. D. Stuurman N. (2014). Advanced methods of microscope control using μManager software. *J. Biol. Methods* 1:e10. 10.14440/jbm.2014.36 25606571 PMC4297649

[B8] Endesfelder U. Malkusch S. Fricke F. Heilemann M. (2014). A simple method to estimate the average localization precision of a single-molecule localization microscopy experiment. *Histochem. Cell Biol.* 141 629–638. 10.1007/s00418-014-1192-3 24522395

[B9] Glebov O. O. Cox S. Humphreys L. Burrone J. (2016). Neuronal activity controls transsynaptic geometry. *Sci. Rep.* 6:22703. 10.1038/srep22703 26951792 PMC4782104

[B10] Glogger M. Spahn C. Enderlein J. Heilemann M. (2020). Multi-color, bleaching-resistant super-resolution optical fluctuation imaging with oligonucleotide-based exchangeable fluorophores. *Angewandte Chemie* 60 6310–6313. 10.1002/anie.202013166 33301653 PMC7986781

[B11] Guo S. M. Veneziano R. Gordonov S. Li L. Danielson E. Perez de Arce K. (2019). Multiplexed and high-throughput neuronal fluorescence imaging with diffusible probes. *Nat. Commun.* 10:4377. 10.1038/s41467-019-12372-6 PMC6763432 31558769

[B12] Hallermann S. Fejtova A. Schmidt H. Weyhersmüller A. Angus Silver R. Gundelfinger E. D. (2010). Bassoon speeds vesicle reloading at a central excitatory synapse. *Neuron* 68 710–723. 10.1016/j.neuron.2010.10.026 21092860 PMC3004039

[B13] Harwardt M. L. I. E. Schröder M. S. Li Y. Malkusch S. Freund P. Gupta S. (2020). Single-molecule super-resolution microscopy reveals heteromeric complexes of MET and EGFR upon ligand activation. *Int. J. Mol. Sci.* 21:2803. 10.3390/ijms21082803 32316583 PMC7215329

[B14] Heilemann M. van de Linde S. Schüttpelz M. Kasper R. Seefeldt B. Mukherjee A. (2008). Subdiffraction-resolution fluorescence imaging with conventional fluorescent probes. *Angewandte Chemie* 47 6172–6176. 10.1002/anie.200802376 18646237

[B15] Jungmann R. Avendaño M. S. Woehrstein J. B. Dai M. Shih W. M. Yin P. (2014). Multiplexed 3D cellular super-resolution imaging with DNA-PAINT and exchange-PAINT. *Nat. Methods* 11 313–318. 10.1038/nmeth.2835 24487583 PMC4153392

[B16] Jungmann R. Steinhauer C. Scheible M. Kuzyk A. Tinnefeld P. Simmel F. C. (2010). Single-molecule kinetics and super-resolution microscopy by fluorescence imaging of transient binding on DNA origami. *Nano Lett.* 10 4756–4761. 10.1021/nl103427w 20957983

[B17] Klevanski M. Herrmannsdoerfer F. Sass S. Venkataramani V. Heilemann M. Kuner T. (2020). Automated highly multiplexed super-resolution imaging of protein nano-architecture in cells and tissues. *Nat. Commun.* 11:1552. 10.1038/s41467-020-15362-1 PMC7096454 32214101

[B18] Kubo A. Misonou H. Matsuyama M. Nomori A. Wada-Kakuda S. Takashima A. (2019). Distribution of endogenous normal tau in the mouse brain. *J. Comp. Neurol.* 527 985–998. 10.1002/cne.24577 30408165 PMC6587864

[B19] Mukherjee K. Yang X. Gerber S. H. Kwon H. B. Ho A. Castillo P. E. (2010). Piccolo and bassoon maintain synaptic vesicle clustering without directly participating in vesicle exocytosis. *Proc. Natl. Acad. Sci. U.S.A.* 107 6504–6509. 10.1073/pnas.1002307107 20332206 PMC2851964

[B20] Nieuwenhuizen R. P. J. Lidke K. A. Bates M. Puig D. L. Grünwald D. Stallinga S. (2013). Measuring image resolution in optical nanoscopy. *Nat. Methods* 10 557–562. 10.1038/nmeth.2448 23624665 PMC4149789

[B21] Ovesný M. Křížek P. Borkovec J. Svindrych Z. Hagen G. M. (2014). ThunderSTORM: a comprehensive imagej plug-in for PALM and STORM data analysis and super-resolution imaging. *Bioinformatics* 30 2389–2390. 10.1093/bioinformatics/btu202 24771516 PMC4207427

[B22] Parthier D. Kuner T. Körber C. (2018). The presynaptic scaffolding protein piccolo organizes the readily releasable pool at the calyx of held. *J. Physiol.* 596 1485–1499. 10.1113/jp274885 29194628 PMC5899986

[B23] Piriya Ananda Babu L. Wang H. Y. Eguchi K. Guillaud L. Takahashi T. (2020). Microtubule and actin differentially regulate synaptic vesicle cycling to maintain high-frequency neurotransmission. *J. Neurosci. Off. J. Soc. Neurosci.* 40 131–142. 10.1523/jneurosci.1571-19.2019 31767677 PMC6939482

[B24] Sarnat H. B. (2013). Clinical neuropathology practice guide 5-2013: markers of neuronal maturation. *Clin. Neuropathol.* 32 340–369. 10.5414/np300638 23883617 PMC3796735

[B25] Sätzler K. Söhl L. F. Bollmann J. H. Borst J. G. G. Frotscher M. Sakmann B. (2002). Three-dimensional reconstruction of a calyx of held and its postsynaptic principal neuron in the medial nucleus of the trapezoid body. *J. Neurosci. Off. J. Soc. Neurosci.* 22 10567–10579. 10.1523/jneurosci.22-24-10567.2002 12486149 PMC6758464

[B26] Sauer M. Heilemann M. (2017). Single-molecule localization microscopy in eukaryotes. *Chem. Rev.* 117 7478–7509. 10.1021/acs.chemrev.6b00667 28287710

[B27] Schindelin J. Arganda-Carreras I. Frise E. Kaynig V. Longair M. Pietzsch T. (2012). Fiji: an open-source platform for biological-image analysis. *Nat. Methods* 9 676–682. 10.1038/nmeth.2019 22743772 PMC3855844

[B28] Schnitzbauer J. Strauss M. T. Schlichthaerle T. Schueder F. Jungmann R. (2017). Super-resolution microscopy with DNA-PAINT. *Nat. Protoc.* 12 1198–1228. 10.1038/nprot.2017.024 28518172

[B29] Schröder M. S. Harwardt M. L. I. E. Rahm J. V. Li Y. Freund P. Dietz M. S. (2020). Imaging the fibroblast growth factor receptor network on the plasma membrane with DNA-assisted single-molecule super-resolution microscopy. *Methods* S1046-2023, 30024–30024. 10.1016/j.ymeth.2020.05.004 32389748

[B30] Schueder F. Stein J. Stehr F. Auer A. Sperl B. Strauss M. T. (2019). An order of magnitude faster DNA-PAINT imaging by optimized sequence design and buffer conditions. *Nat. Methods* 16 1101–1104. 10.1038/s41592-019-0584-7 31591576

[B31] Shahmoradian S. H. Lewis A. J. Genoud C. Hench J. Moors T. E. Navarro P. P. (2019). Lewy pathology in Parkinson’s disease consists of crowded organelles and lipid membranes. *Nat. Neurosci.* 22 1099–1109. 10.1038/s41593-019-0423-2 31235907

[B32] Smith C. S. Joseph N. Rieger B. Lidke K. A. (2010). Fast, single-molecule localization that achieves theoretically minimum uncertainty. *Nat. Methods* 7 373–375. 10.1038/nmeth.1449 20364146 PMC2862147

[B33] Spahn C. Grimm J. B. Lavis L. D. Lampe M. Heilemann M. (2019a). Whole-cell, 3D, and multicolor STED imaging with exchangeable fluorophores. *Nano Lett.* 19 500–505. 30525682 10.1021/acs.nanolett.8b04385

[B34] Spahn C. Hurter F. Glaesmann M. Karathanasis C. Lampe M. Heilemann M. (2019b). Protein-specific, multicolor and 3D STED imaging in cells with DNA-labeled antibodies. *Angewandte Chemie* 58 18835–18838. 10.1002/anie.201910115 31603612 PMC6972974

[B35] Strauss S. Jungmann R. (2020). Up to 100-fold speed-up and multiplexing in optimized DNA-PAINT. *Nat. Methods* 17 789–791. 10.1038/s41592-020-0869-x 32601424 PMC7610413

[B36] Venkataramani V. Kardorff M. Herrmannsdörfer F. Wieneke R. Klein A. Tampé R. (2018). Enhanced labeling density and whole-cell 3D dSTORM imaging by repetitive labeling of target proteins. *Sci. Rep.* 8:5507. 10.1038/s41598-018-23818-0 PMC5882651 29615726

[B37] Verstreken P. Ly C. V. Venken K. J. T. Koh T. W. Zhou Y. Bellen H. J. (2005). Synaptic mitochondria are critical for mobilization of reserve pool vesicles at *Drosophila* neuromuscular junctions. *Neuron* 47 365–378. 10.1016/j.neuron.2005.06.018 16055061

[B38] Wang Y. Woehrstein J. B. Donoghue N. Dai M. Avendaño M. S. Schackmann R. C. J. (2017). Rapid sequential in situ multiplexing with DNA exchange imaging in neuronal cells and tissues. *Nano Lett.* 17 6131–6139. 10.1021/acs.nanolett.7b02716 28933153 PMC5658129

[B39] Zorgniotti A. Ditamo Y. Arce C. A. Bisig C. G. (2021). Irreversible incorporation of L-Dopa into the C-Terminus of α-tubulin inhibits binding of molecular motor KIF5B to microtubules and alters mitochondrial traffic along the axon. *Neurobiol. Dis.* 147:105164. 10.1016/j.nbd.2020.105164 33171229

